# The Interaction Between StCDPK14 and StRbohB Contributes to Benzo-(1, 2, 3)-Thiadiazole-7-Carbothioic Acid S-Methyl Ester-Induced Wound Healing of Potato Tubers by Regulating Reactive Oxygen Species Generation

**DOI:** 10.3389/fpls.2021.737524

**Published:** 2021-11-15

**Authors:** Li Ma, Hong Jiang, Yang Bi, Yong-Cai Li, Jiang-Wei Yang, Huai-Jun Si, Ying-Yue Ren, Dov Prusky

**Affiliations:** ^1^College of Horticulture, Gansu Agricultural University, Lanzhou, China; ^2^College of Food Science and Engineering, Gansu Agricultural University, Lanzhou, China; ^3^College of Life Science and Technology, Gansu Agricultural University, Lanzhou, China; ^4^Department of Postharvest Science, Agricultural Research Organization, Rishon LeZion, Israel

**Keywords:** potato tuber, wound healing, StCDPK14, StRbohB, ROS

## Abstract

Reactive oxygen species (ROS) production is essential for both physiological processes and environmental stress in diverse plants. Previous studies have found that benzo-(1, 2, 3)-thiadiazole-7-carbothioic acid S-methyl ester (BTH)-inducible ROS were associated with wound healing of potato tubers. Calcium-dependent protein kinases (CDPKs), the important calcium receptors, are known to play a crucial part in plant development and adaptation to abiotic stresses. However, whether CDPK-mediated ROS generation induced by BTH is involved in wound healing is elusive. In this study, we measured *Solanum tuberosum CDPKs* (*StCDPKs*) expression using real-time PCR, and it was found that the transcriptional levels of *StCDPKs* from BTH-treated tissues were significantly induced, among which *StCDPK14* presented the most increased level. Subcellular localization results showed that StCDPK14 is located in the nucleus and membrane. The transgenic potato plants and tubers were developed using interference-expression of *StCDPK14* by *Agrobacterium tumefaciens*–mediated transformation. The *St* respiratory burst oxidase homologs (*StRbohs*) expression showed a remarkable decrease in *StCDPK14* transgenic tubers, notably, H_2_O_2_ content and suberin deposition were also significantly declined. To confirm the relationship between StCDPK14 and StRbohB, yeast-two-hybrid and bimolecular fluorescence complementation were used to examine the interaction, and it was shown that StCDPK14 interacted with the specific Ca^2 +^ -binding motif (helix-loop-helix, called EF-hand) of StRbohB N-terminus. The above results unraveled that StCDPK14 functions in ROS generation *via* interacting with StRbohB during wound healing of potato tubers.

## Introduction

Wound healing is a typical characteristic of harvested potato tubers, which protects against pathogen infection and prevents water evaporation (Lulai et al., 2016). The optimal healing conditions of potato tubers are approximately 20°C with relative humidity (RH) of 80–100%. However, the harvested potato tubers in fall easily suffer from cold stress and the healing is markedly slower (Voss, 2016), therefore, it is necessary to determine the measures and related mechanism to accelerate the progress of wound healing. Our previous study indicated benzo-(1, 2, 3)-thiadiazole-7-carbothioic acid S-methyl ester (BTH), an analog of salicylic acid (SA) and also the first artificially synthesized and commercialized elicitor registered as Bion^®^ or Actigard^®^, stimulates defense responses *via* reactive oxygen species (ROS) production in diverse plants and promotes the wound healing of potato tubers by accelerating deposition of suberin and lignin at wound sites (Jiang et al., 2019). Further research has demonstrated the elicited wound healing of potato tubers by BTH involves in ROS metabolism *via* an increase of the respiratory burst oxidase homolog (Rboh) activity and transcriptional levels, leading to the enhancement of ROS (Jiang et al., 2020). ROS appears to contribute to the polymerization of phenolic monomers in suberin synthesis, and also to the upregulation of defense-related genes as a signaling molecule (Tenhaken et al., 1995; Kumar et al., 2007). There are several pathways to produce necessary ROS for wound healing of potato tubers; the Rboh is a major one (Razem and Bernards, 2003). Rbohs that are found in the plasma membrane are key regulators of ROS production (Yoshioka et al., 2003), and play pleiotropic roles in developmental processes and was required for certain wound response expression in *Lycopersicon esculentum* (Sagi et al., 2004). Additionally, evidence demonstrated that the wound-induced oxidative burst of superoxide mediated by *StRbohA* promotes the wound healing of potato tubers (Kumar et al., 2007).

The respiratory burst oxidase homologs (Rbohs) carry an extension comprising two EF-hand motifs at N-terminus, indicating that Ca^2+^ might activate its activity *via* a directly calcium binding (Liu and He, 2016). The ROS-producing activity of Rboh induced by Ca^2+^ is an early event during the plant defense response (Lecourieux et al., 2006; Zhang et al., 2014). As a second messenger, Ca^2+^ is an essential component that affects protein kinase signaling pathways (Klimecka et al., 2011). Calcium-dependent protein kinase (CDPK) is one of the major Ca^2+^ sensors found in plants, and also a class of serine (Ser)/threonine (Thr) protein kinases that have a conserved structure (Kolukisaoglu et al., 2004). The CDPK comprises four typical domains, including a variable N-terminal domain, a Ser/Thr kinase domain, an auto inhibitory junction region, and a calmodulin-like domain (CaM-LD) harboring EF-hand motifs at the C-terminal region (Cheng et al., 2002; Harmon, 2003). It has been suggested that the variable N-terminal domain contains potential myristoylation or palmitoylation sites that are associated with subcellular targeting, which determine the function of CDPK (Hrabak et al., 2003; Asai et al., 2013). Ca^2+^ binding in response to environmental changes alters the conformational structure, leading to an indirectly activation of the kinase to phosphorylate downstream target proteins such as Rbohs (Liu J. Y. et al., 2017).

Calcium-dependent protein kinase serves as the upstream element of Rboh to produce ROS by phosphorylation events, which has a critical role in signaling pathways (Giammaria et al., 2011). Earlier works demonstrated that Rboh is one of the potential substrates for CDPK in defense against pathogen attack, and its activity is activated by phosphorylation of the N-terminal region, suggesting that transcriptional and post-translational events of Rbohs stimulate an oxidative burst in potato (Kobayashi et al., 2007; Giammaria et al., 2011). StRbohB in potato was activated by StCDPK5 to regulate oxidative burst in responses to *Phytophthora infestans* infection (Kobayashi et al., 2012). In *Arabidopsis*, *AtCPK5*/*AtCPK6* and *AtCPK4*/*AtCPK11* are also found to regulate ROS generation (Boudsocq et al., 2010), and *AtCPK5* has been demonstrated to interact with *AtRbohD* and facilitate rapid signal propagation for defense response activation (Dubiella et al., 2013). In turnip, the interaction of *BrrRbohD1* with *BrrCDPK10* and *BrrRbohD2* with *BrrCDPK4/7/9/10/17/22/23* involves in H_2_O_2_ accumulation and resistance against *pst* DC3000 infection (Wang et al., 2017). In *Nicotiana benthamiana*, *NbCDPKiso2* activated *NbRBOHB* to trigger ROS accumulations under viral infection (Hyodo et al., 2017). However, most of the oxidative burst mediated by *CDPKs* is triggered by biotic stresses, the function and mechanism of *CDPKs* induced by abiotic stress such as wounding in plants remain elusive (Atif et al., 2019), especially under the action of resistance inducer.

While BTH elicited ROS production of potato tubers during healing has been studied, little information is known on the effect of BTH on Ca^2+^ concentration, expression patterns of CDPKs, and even the regulation between CDPK and Rboh. In this article, the Ca^2+^ distribution, Ca^2+^ concentration, and CDPKs expression in potato tubers treated with BTH treatment were analyzed, a CDPK isoform of *StCDPK14* (PGSC0003DMG400009883) was characterized, and transgenic potato plants and tubers were generated using interference-expression of *StCDPK14*. Meanwhile, the role and the possible mechanism of *StCDPK14* involvement in wound healing were investigated by analyzing the H_2_O_2_ production and suberin deposition in transgenic tubers, together with an assay of the interaction between StCDPK14 and StRbohB by yeast-two-hybrid and bimolecular fluorescent complimentary (BiFC).

## Materials and Methods

### The Seed Potatoes and Potato Plantlets

The seed potatoes were purchased from Gansu Ailan Potato Seed Industry Co. Ltd. The potato plantlets “*Solanum tuberosum* L. cv. Atlantic” and the tobacco plant (*N. benthamiana* L.) were provided by the Molecular Biology Laboratory of College of Life Science and Technology in Gansu Agricultural University, where the experiment was carried out from April to October 2019.

### Growth Conditions of Plant Materials

The potato plantlets were propagated by subculturing using single-node cuttings on Murashige and Skoog (MS) basal medium containing 3% sucrose and 0.45% agar and grown in an illuminating incubator providing a light: dark regimen of 16: 8 h and a light intensity of 20000lx at 25 ± 2°C. Micro-tubers were screened and multiplicated on MS media containing 8% sucrose and 0.45% agar under dark conditions at 25 ± 2°C. Tobacco plants were cultured in an environmentally controlled growth chamber with a 16 h light/8 h dark cycle at 25°C. The relative humidity was maintained at 60–70% and was used for subcellular location analysis.

### Wound Healing and Sampling of Potato Tubers

The potato tubers used for wound healing were washed and stored at 5°C for further analysis. The tubers of uniform size and without injury were wounded and healed after BTH treatment according to the method described by Jiang et al. (2019). Healing tissues samples (2 mm depth) were collected from the wounded surface after healing for 0, 1, 3, 5, 7, and 14 days. All the samples were frozen in liquid nitrogen and stored at −80°C for subsequent experiments.

### Distribution of Cellular Ca^2+^ in Potato Tuber Healing Tissue

The distribution of cellular Ca^2+^ in healing tissues was based on the method described by De Freitas et al. (2012) with some modification. The tissue blocks of 1 mm^3^ cut from the healed region were incubated in 2.5% glutaraldehyde and sucked to vacuum. Then, the tissues were rinsed using 0.1 M sodium cacodylate trihydrate buffer containing 2% potassium antimonite five times, each time for 4 h at 4°C, the tissues were post-fixed in 1% osmic acid for 2 h, and washed 5 min again by sodium cacodylate trihydrate buffer. Then, the tissues were dehydrated in ethanol with various concentration gradients and embedded in epoxy resin. 1–2 μm sections were prepared and dyed with uranium acetate and lead citrate. For observation of Ca^2+^, the transmission electron microscope (TEM) (Leica SP8, Germany) was used.

### Observation of Cytosolic Ca^2+^ Concentration in Potato Tuber Healing Tissue

The presence of Ca^2+^ in the cytosolic was determined *via* staining with Fluo-3-acetoxymethyl ester (Fluo-3-AM), according to the protocol described by Markulin et al. (2019). The sections of healing tissues (0.3–0.5 mm) at 4 and 72 h were incubated in 10 μM Fluo 3-AM for 24 h at 4°C, washed twice with phosphate-buffered saline and examined with a fluorescence microscope (BX61 LSM 800, Olympus, Japan) using excitation filter at 488 nm and emission filter at 515–565 nm. Fluorescent pictures of cytosolic Ca^2+^ were obtained under 10× magnification.

### Real-Time Quantitative PCR Analysis in Potato Tuber Wound-Healing Tissue

Total RNA was isolated from the transgenic tubers using a simple Total RNA Kit (Cat. No. DP419, TIANGEN208 Biotech, China). The RNA integrity was determined using 1% agarose gel, the concentration and purity were established at an absorbance of 260 nm and a 260/280 ratio, respectively. First-strand cDNA synthesis was reverse transcribed using the TIAN script RT Kit211 (Cat. No. KR116, TIANGEN Biotech, China) according to the manufacturer’s instructions.

The obtained cDNA was used in an expression assay of *StCDPKs* by real-time quantitative PCR (qRT-PCR) on the Light Cycler 96 SW 1.1 instrument. The cDNA concentration of the transcript was measured and diluted to 100 ng/μL as a template for qRT-PCR. The qRT-PCR reaction consisted of 1 μL cDNA template (ca.0.1 μg cDNA), 10 μL 2× Super Real PreMix Plus (with SYBR Green), 0.4 μL 50× ROX Reference Dye, 0.6 μL primers, and 7.4 μL RNase-Free ddH_2_O. The elongation factor 1-alpha 1 [*ef1a*, (NM_001273486.1)] was used as an internal control gene. qRT-PCR was performed with the following conditions: 94°C for 900 s, with 1 cycle; 95°C for 30 s, 55°C for 20 s with 40 cycles, and finally an extension step for 30 s at 72°C. The relative expressional levels of each gene were calculated using the 2^–ΔΔ*C(t)*^ method compared to that of 0 h (Livak and Schmittgen, 2001). Primer sequences used for RT-qPCR are shown in Table 1.

**TABLE 1:** Primer sequences and efficiencies for real-time quantitative (qRT)-PCR expression analyses of target genes involved in tuber wound healing.

**Name of primer**	**NCBI gene ID**	**Primer sequence (5′ → 3′)**	
		**Forward**	**Reverse**
*StCDPK1*	NM_001288393	GGTGGAGTTGGGGGTAAAGG	ATTGAGTTTCTGGGCCTGGAG
*StCDPK2*	XM_006346152	TGAAGTGGACACGGACAATG	GACCTTGCCTGGTTGCTTG
*StCDPK3*	NM_001288527	CTGCTCAGTGGTGTACCTCC	TTCCTTGGCTCCTGCGTTAG
*StCDPK4*	NM_001287877	TCCCACCAGTAACGCTCAAC	AGTCCCAAACTGCCCTTGTC
*StCDPK5*	NM_001287861.1	CTGCGGGTGATTGCTGAAAG	CCGCATCCATAAGTTCCCGT
*StCDPK6*	XM_006345687	CTGCGAGGCAAACTAGATTTAG	CCACGGATGGCACAAAACTT
*StCDPK7*	NM_001318643	TCGCCGGATGATAGTGCTTC	TGTCATCTGTTCTGGTGGCT
*StCDPK8*	XM_006366477	TAAACATGCCTCTGGGAGTGG	TCCGAGCTCACGACCCAAAT
*StCDPK9*	XM_006348373	TGCACGCCAACAAAATCGAG	ACCGAATTCCTCACAAGCCT
*StCDPK10*	XM_006351162	GCACCTGAAGAAGGCGTTTG	CCGTCCATCCTTGTCAGTGT
*StCDPK11*	XM_006353564	AGGGTCTGACCTAGTGGAGTC	CCGTAGTCAATCGTGCCACT
*StCDPK12*	XM_006339117	TTGGGGTGAAAGTGAGCAGG	CTCGGGTCCCTAACAAGCAT
*StCDPK13*	XM_006364680	AATACATGCTCCGGACCCAC	ATGTGGAGGAGGGGTGTTCT
*StCDPK14*	XM_006342017	GGGCTGAGACTGAAAAGGGC	TGTTGGAGGGCTTCATCTGC
*StCDPK15*	XM_006351851	CACCAGGGATCCTAGAGCAC	GGGTCTGTCTGGAGCAACAT
*StCDPK16*	XM_006343307	ACATGGTTTGGTGCATCGTG	TCCCTGGTCTGATGAAGTCTG
*StCDPK17*	XM_006356324	CCATGGCCTGCAATTTCACAT	GGTGCATCTCCATCCTCCTTG
*StCDPK18*	XM_006349733	CACACAAACAAACAGGGGAGC	ACCTCCAGCACACAATTCCAT
*StCDPK19*	XM_006352199	CCACCTCCACGACCATTCTC	ATTGACTTGCACGCGAACTG
*StCDPK20*	XM_006348361	GGGAGCTTTTCGACAGGAT	GGGCGAATCTTCATCCTGGT
*StCDPK21*	XM_006339122	GAAAGGCGCGGTGGATAGAT	TGTTTCACCCCTTCCACAGG
*StCDPK22*	XM_006340676	ACCCCTTCCACCACCAATAC	ACCCCACCGTTATCCTTACC
*StCDPK23*	XM_006347224	GGGACAAACTGTTGCTGAACC	ACCTTTGTAAGTGCACAGCC
*StRbohA*	NM_001288375.1	GTTTACCTGGGCATGAACGC	CTCCACCAATACCGACTCC
*StRbohB*	NM_001288052.1	GGTTTACCTGGGCATGAACG	TACAGTAGCCGGTTCAACGC
*StRbohC*	NM_001288524.1	TGTCTTGCTAAGGGTGCTG	ACCACCAATAGCTTTCGG
*StRbohD*	NM_001318578.1	AGCCCCAATTCAACCAGATG	CAGTACCCAAACTCTTCGCC
*StRbohE*	XM_006363326.2	TTGAAGGAACGTGCAGCC	ATCCAGCCTCTTTGCCAGT
*StRbohH*	XM_006353710.2	GGTTCTAGTGATGAGTGCTGC	GCCCATCTTCTGATCCAACCAT
*efla*	AB061263	ATTGGAAACGGATATGCTCCA	TCCTTACCTGAACGCCTGTCA

### Bioinformatics Analysis of StCDPK14

The full-length cDNA sequence of StCDPK14 was obtained from the National Center for Biotechnology Information^1^ with *StCDPK14* as a query (XM_006342017.2). The number of EF-hand Ca^2+^ binding structures was predicted using the simple modular architecture research tool (SMART) program.^2^ The prediction of myristoylation and palmitoylation sites was performed by myristoylator^3^ and CSS-Palm3.0,^4^ respectively. The conserved domain analysis was performed using SMART (see text footnote 2). Prediction of interacting proteins with StCDPK14 was constructed by the search tool for the retrieval of interacting genes/proteins (STRING) software.^5^

### Subcellular Localization of StCDPK14

The coding sequences of *StCDPK14* gene without a stop codon were amplified by PCR and subcloned into the pEGFP vector, in frame with the GFP sequence, resulting in StCDPK14-GFP vectors under the control of the CaMV 35S promoter. The primers used are listed in Table 2. The GFP fusion construct was mixed with the membrane and nucleus marker and co-transformed into *N. benthamiana* leaves by *Agrobacterium tumefaciens* infiltration. The leaf discs near the injection site were cut 48 h after infiltration and the lower epidermis was selected to observe signals of GFP. Fluorescence signals were visualized at 488 nm and detected under a confocal laser scanning microscope (Leica SP8, Germany).

**TABLE 2:** Primer sequences used in subcellular localization, transgenic plant, yeast-two-hybrid, and bimolecular fluorescent complimentary analysis.

**Name of primer**	**Primer sequence (5′ → 3′)**
pEGFP-StCDPK14	Forward: ACTCTTGACCATGGTGAAGATCTCCAATGGGTCTCTGTTTTAC Reverse: ATCCTAGGACTAGTCGAAGATCT TCTTGGCTTCACTTCATC
pHellsgate8-StCDPK14	Forward: GGGGACAAGTTTGTACAAAAAAGCAGGCTGCGCAAGTATGGGAAGGAGA Reverse: GGGGACCACTTTGTACAAGAAAGCTGGGTTAAGCCACGGATGTTGGAGG
pGBKT7-CDPK14	Forward: ATGGAGGCCGAATTCATGGGTCTCTGTTTTACT Reverse: TGCAGGTCGACTCATCTTGGCTTCACTTCAT
pGADT7-RbohB	Forward: ATGGAGGCCAGTGAATTCATGGAGATCGAAAAC Reverse: ATGCCCACCCGGGTGGAATTCTTAGAAATTTTCTTT
PE3308-CDPK14	Forward: TCGAGCTCAAGCTTCGAATTCCCAATGGGTCTCTGTTTTAC Reverse: GTACCGTCGACTGCAGAATTCTCTTGGCTTCACTTCATC
PE3349-RbohB	Forward: GAGCTCAAGCTTCGAATTCCGGGAAAATCAAATGG Reverse: CGGTACCGTCGACTGCAGAATTCGAAATTTTCTTTATG
CDPK14-YN	Forward: ACAAGTTTGTACAAAAAAATGGGTCTCTGTTTTACTAAAG Reverse: CACCACTTTGTACAAGAATCTTGGCTTCACTTCATCAAC
RbohB-YC	Forward: ACAAGTTTGTACAAAAAAATGGAGATCGAAAACACGA Reverse: CACCACTTTGTACAAGAACTTAAGTTTCTGACTGAGC

### Creation of Transgenic Potato Plants and Molecular Verification

The *StCDPK14* coding sequence was amplified using the primers listed in Table 2, the product was then cloned into the pHellsgate8 vector using gateway cloning technology and resulted in an interference-expression (pHellsgate8-*StCDPK14*) construct that was transformed into *A. tumefaciens* LBA4404 according to the method described by Zhang et al. (2018). The potato tubers obtained from sub-culture were removed as buds and cut into slices of 1–2 mm, and were infected by *Agrobacterium* containing pHellsgate8-*StCDPK14* and the empty pHellsgate8 plasmid. The infected slices were placed on MS solid media at 28°C in the dark for 48 h and after that, they were transferred into differentiation media for culture in a light chamber (16 h light/8 h dark with a light intensity of 20,000lx) at 25°C. When the new buds were generated from the center callus of the potato slice, they were transferred into rooting MS medium supplemented with 75 mg/L kanamycin and 200 mg/L carbenicillin for screening kanamycin-resistant transformed plants. After 1–2 months, the regenerated plantlets were acclimatized and grown in flasks under the condition of a photoperiod of 16/8 h light/dark at 25°C.

The genomic DNA of the transgenic plants was isolated using a plant genomic DNA isolation kit (Cat. No. DP305, TIANGEN Biotech, China). The kanamycin-resistant potato plants were screened using the neomycin phosphate (NTP II) gene with a pair of primers to detect positive transformations of the *StCDPK14* transgenic lines. The DNA from wild-type potato plants was used as a negative control and the pHellsgate8-*StCDPK14* as the experimental set. A PCR was performed as described in the above section. The positive and rooting plants were chosen for further culture and transgenic tubers were obtained after approximately 3 months of growth. In this experiment, the transgenic tubers from three interference-expression lines were mixed and used for transcript level of *StCDPK14* and StRbohs and ROS content. The transgenic tubers from the line of *StCDPK14-D* were only used for the observation of suberin deposition.

### Assay of O_2_^–^ and H_2_O_2_ Content in Transgenic Tuber Tissue at Wound Sites

The measurement of O_2_^–^ and H_2_O_2_ content in healing tissues was performed using the commercial kits (Suzhou Comin Biotechnology Co. Ltd.) according to manufacturer’s instruction. For O_2_^–^, 0.1 g healed tissue was homogenized in extracted solution, centrifuged at 12,000 rpm for 20 min, and then the supernatants were mixed with four kinds of solution. After centrifugation of the mixture at 8000 rpm, the supernatants were prepared for measurement. For H_2_O_2_, 0.1 g healed tissue was homogenized in 1 mL acetone and centrifuged at 8000 × *g* at 4°C for 10 min. The supernatants were removed and added into a reaction solution, incubated for 5 min at room temperature and used for determination. The absorbance of the reaction to determine O_2_^–^ and H_2_O_2_ content was measured at 415 and 530 nm, respectively. The O_2_^–^ and H_2_O_2_ content were calculated and expressed as μmol⋅g^–1^ FW and nmol⋅g^–1^ FW, respectively.

### Suberin in Wound-Healing Tissues of Transgenic Tubers

The suberin deposition in transgenic tuber wound-healing tissue was microscopically detected by staining with toluidine blue and neutral red according to the method of Jiang et al. (2019). Six tubers of transgenic and wild-type control were used to observe the suberin deposition using a microscope (BX53, Olympus, Japan).

### Yeast-Two-Hybrid of StCDPK14 With StRbohB

The yeast-two-hybrid analysis was conducted according to the Matchmaker Gold Yeast-Two-Hybrid System User Manual. Full length StCDPK14 was inserted into the pGBKT7 (GAL4 DNA-binding domain cloning vector) bait plasmid (pGBKT7-CDPK14), and the coding region of StRbohB was cloned into the vector of pGADT7 (GAL4 activation domain cloning vector) (pGADT7-RbohB). Both plasmids were then co-transformed into the yeast strain Y2HGold. Primers used in this assay are listed in Table 2. Mediums lacking Leu-Trp and Leu-Trp-His were used for selecting positive interactions.

### Bimolecular Fluorescent Complimentary of StCDPK14 With StRbohB and EF-Hand Motifs of StRbohB

A BiFC assay was conducted as described by Zhou et al. (2018). The coding region of StCDPK14 was cloned into the pSAT1-nVenus-N (PE3308) vector, resulting in nVenus-StCDPK14, and the coding sequence of StRbohB was cloned into pSAT1-cCFP-N (PE3449), resulting in StRbohB-cCFP. Primers used are listed in Table 2. Transient expression of protoplasts was detected *via* the polyethylene glycol-mediated transformation method. Confocal laser scanning microscope (Olympus FV 1000, Japan) was used to visualize fluorescence.

In addition, the coding region of StCDPK14 was cloned into the pEarleyGate201 vector, the EF-hand motifs of StRbohB were cloned into the pEarleyGate202 vector, resulting in pEarleyGate201-CDPK14-YN and pEarleyGate202-RBOHB-YC. The plasmid constructs were expressed in *N. benthamiana* leaves by *Agrobacterium* infiltration. The fluorescence was then visualized by a confocal laser scanning microscope (LeciaSP8, Germany). Primers used are listed in Table 2.

### Statistical Analysis

All the above experiments above were performed in triplicate. Data are expressed as the means (±) of three biological replicates in each treatment. Statistical significance was examined using the least significant difference (LSD) when *P* < 0.05 with statistical product and service solutions (SPSS) 21.0 software. All the charts were drawn using OriginPro 8.5.

## Results

### Effect of Benzo-(1, 2, 3)-Thiadiazole-7-Carbothioic Acid S-Methyl Ester Treatment on Cellular Ca^2+^ Distribution and Ca^2+^ Levels in Healing Tissues of Potato Tuber

**FIGURE 1:**
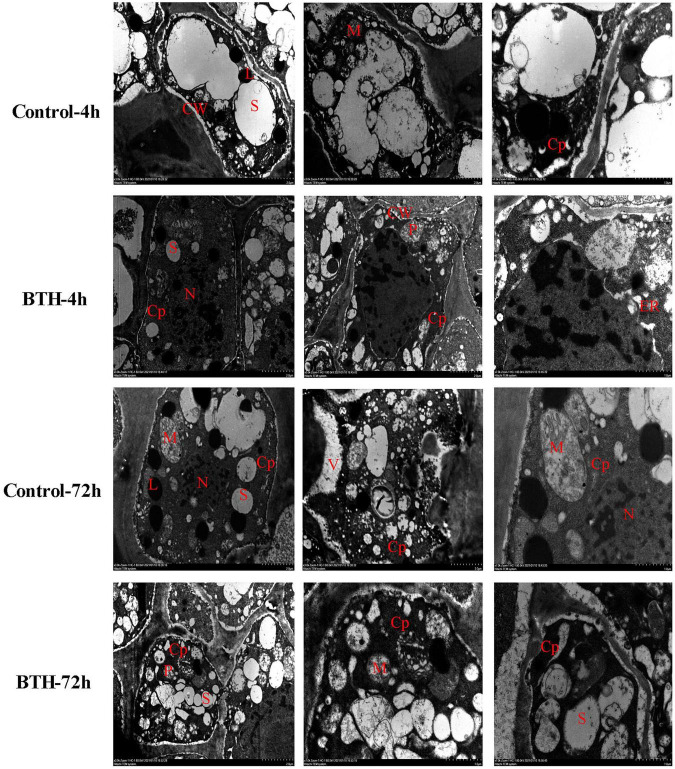
The cellular Ca^2+^ distribution in healing tissues of potato tubers. Black spots represent Ca^2+^ pyroantinonate precipitate particles. S: starch; Cp: cytoplasm; CW: cell wall; N: nucleus; M: mitochondria; L: lipids; ER: endoplasmic reticulum; V: vacuole.

The TEM observation showed that Ca^2 +^ precipitate particles in control and BTH-treated healing tissues distributed in large quantity in cytoplasm, and occasionally in cellular Ca^2 +^ sink, such as mitochondria, endoplasmic reticulum, vacuoles, and plasmids. Additionally, large amounts of Ca^2+^ distribution were also found in the nucleus (Figure 1). However, it is not sure whether the cellular Ca^2+^ concentration was elevated by BTH treatment. Therefore, the further observation of cellular Ca^2+^ concentration by Fluo-3-AM was performed.

**FIGURE 2:**
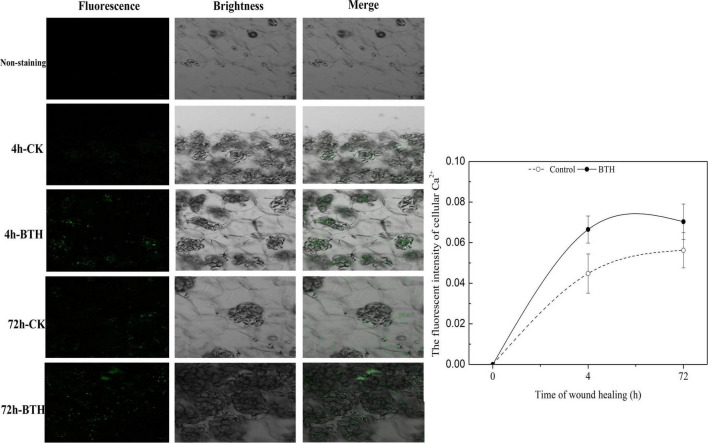
Fluorescence of cytosolic Ca^2+^ in benzo-(1, 2, 3)-thiadiazole-7-carbothioic acid S-methyl ester (BTH)-treated tissues of potato tuber at 4 and 72 h of healing. Bar = 50 μm.

To evaluate the effect of BTH treatment on Ca^2+^ concentration at 4 and 72 h of wound healing of potato tubers, the fluorescence intensity that stained with Fluo-3-AM was further visualized on the wounded tubers. The results showed that the fluorescent granules in BTH-applied and control tubers were clearly observed, whereas the non-stained controls did not show fluorescence. After 4 or 72 h of wound healing, the fluorescence of cytosolic Ca^2+^ levels in BTH treatment was increased compared to that in the control, indicating that BTH markedly increased Ca^2+^ levels in healing tissue of potato tuber (Figure 2).

### Effect of Benzo-(1, 2, 3)-Thiadiazole-7-Carbothioic Acid S-Methyl Ester Treatment on the Transcript Levels of *StCDPKs* in Healing Tissue of Potato Tubers

**FIGURE 3:**
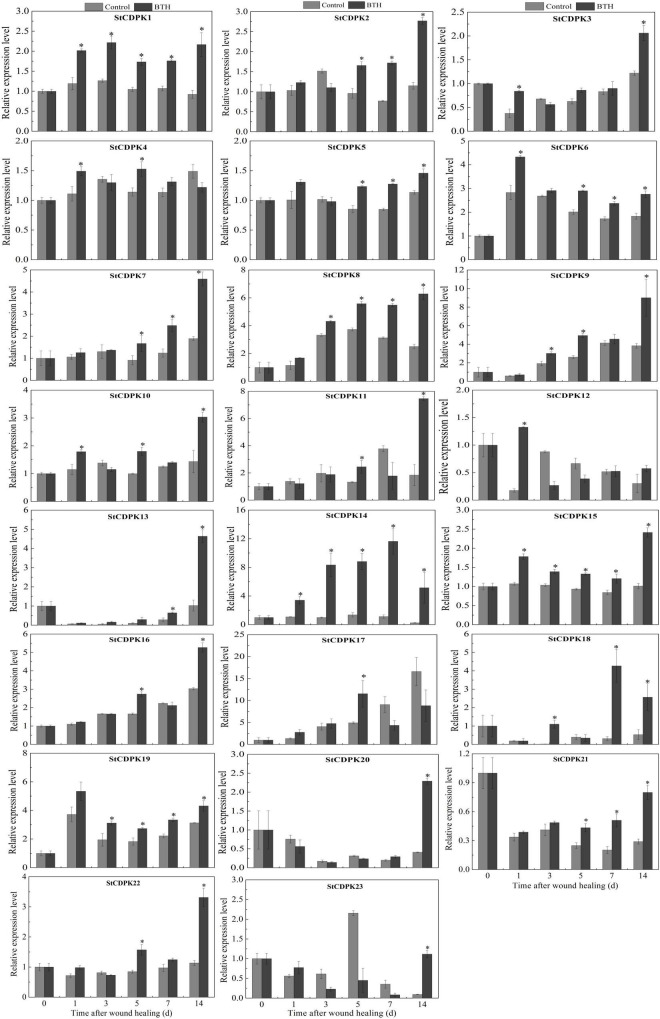
Expression levels of *Solanum tuberosum calcium-dependent protein kinases* (*StCDPKs*) (*StCDPK1-StCDPK23*) in healing tissues of tubers treated with BTH. The expression data at each time point is relative to that of 0 d (untreated tubers), which was set to 1. The mean (±SD) represents the value of three replicates. Asterisks indicate statistical significance (*P* < 0.05).

A total of 23 CDPK genes in tubers treated with BTH were isolated and their expression profiles in healing tissues were assessed by qRT-PCR (Figure 3). During the early stage of healing (0–1 day), *StCDPK1/3/4/5/6/10/12/15/23* were BTH-inducible, whereas the others were not affected by the elicitor. The expression of *StCDPK4* and *StCDPK10* in BTH-treated tissues both showed a peak on the first day of healing. During the middle and late stages of healing (3–14 days), *StCDPK1/8/9/10/14/15/18/19* were upregulated in BTH-treated tissues. Moreover, the *StCDPK2/5/6/7/21* were only increased by BTH during the late stage of healing (5–14 days), and they were observed to be gradually increased except for *StCDPK*6. *StCDPK12* was not observed to be BTH-inducible during the late stage of healing.

Among these genes, *StCDPK14* was induced significantly by BTH in comparison with other members, which was 3.1-fold, 8.3-fold, 6.4-fold, 10-fold, and 20-fold of the control after 1, 3, 5, 7, and 14 days of healing, respectively. Additionally, according to the RNA sequencing analysis of healing tissues treated with BTH, *StCDPK14* was similarly induced (Supplementary Table 1), where the fragments per kilobase of exon model per million reads mapped value (FPKM) was also found to be upregulated the most, with an increase of 4.1-fold in comparison with the control. These data suggested that *StCDPK14* might play a critical role during wound healing induced by BTH. Therefore, the *StCDPK14* was selected for further experiments to reveal the molecular mechanism.

### The Information Acquisition and the Subcellular Location of the StCDPK14 Protein

**FIGURE 4:**
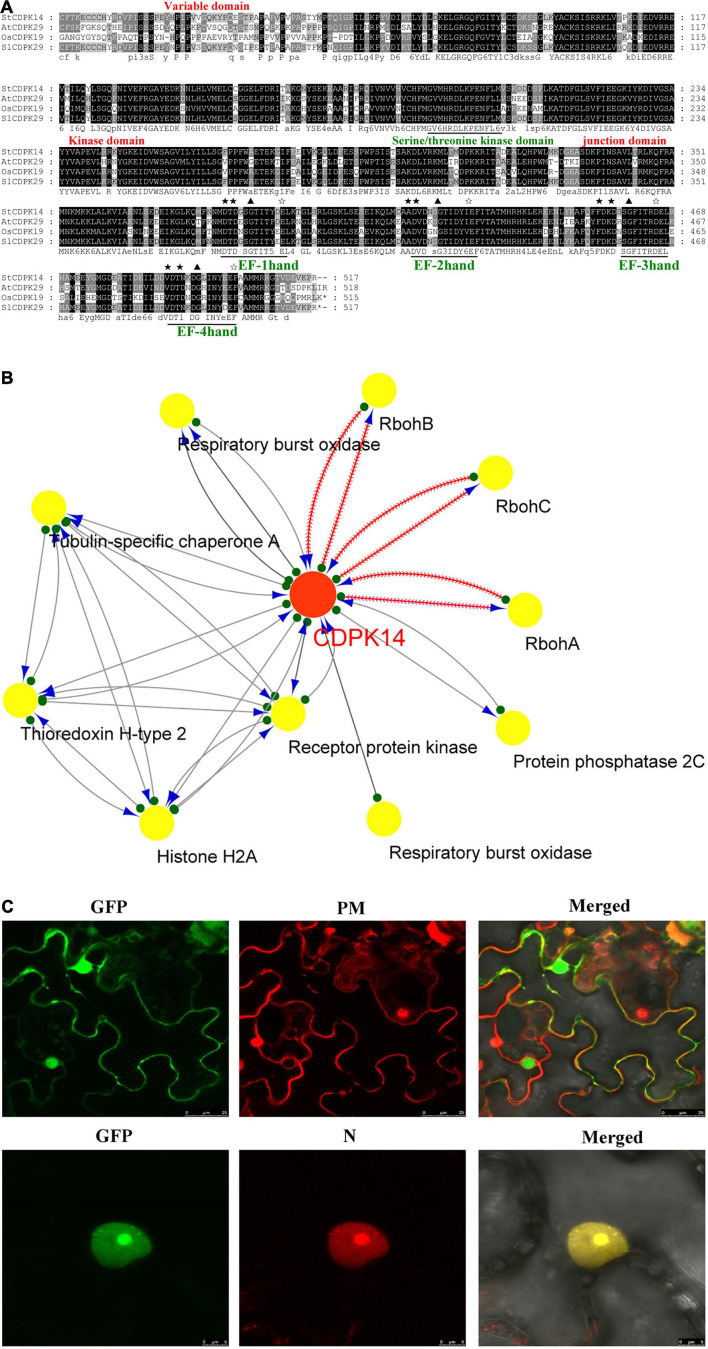
Alignment of StCDPK14 and the predicted protein interaction network predicted for StCDPK14 and subcellular localization of StCDPK14-GFP. **(A)** Amino acid sequence alignment of *StCDPK14* with *AtCDPK29* (NP_974150.2), *SlCDPK29* (XP_004238385), and *OsCDPK19* (XP_015646656.1). Fully conserved resides are highlighted with a black background and 75% conserved residues by a gray background. **(B)** Protein interaction network of StCDPK14 and StRbohs in potato. Red node presents the input protein and yellow nodes are the predicted interactors. **(C)** Subcellular localization of StCDPK14-GFP fusion proteins in *N. benthamiana* leaf epidermal cells. The left panel represents GFP fluorescence, the middle represents membrane and nucleus marker, and the right is a merge of the two images. Bar = 25 μm. ★ Represent the core sequences and the conserved D-x-D residues in four EF-hand, ✰ represent the conserved sequences E-E-L-K, E-F-I-T, D-E-L, and E-F-A/V-A-M-M that is rich in Glu (E) after EF-hand domian, respectively.

The information acquisition related to proteins based on bioinformatics analysis could provide an important foundation for further functional dissection of potato CDPKs. Comparison of the sequence of the StCDPK14 protein with those from other species including *Arabidopsis thaliana*, *Solanum lycopersicum*, and *Oryza sativa* in the GenBank and Phytozome databases indicated that it shared a significant similarity with *AtCDPK29* (90%), *SlCDPK29* (89%), and *OsCDPK19* (88%), respectively (Figure 4A). They shared the conserved variable domain at the N-terminal, Ser/Thr kinase domain, junction domain, and four EF-hands motif domains, suggesting that StCDPK14 was equipped with the complete domain structure of a kinase protein. Moreover, StCDPK14 was predicted to interact with StRbohA, StRbohB, and StRbohC (Figure 4B), therefore, it was tempting to speculate that an interaction between them might occur.

The different and specific subcellular locations of CDPKs may provide the potential for isoform-specific differences in mediating diverse cellular functions. To detect the subcellular localization of StCDPK14, the fusion protein of StCDPK14-eGFP was created and transformed into *N. benthamiana* leaves *via* the *A. tumefaciens* mediated method. Confocal micrographs displayed that the StCDPK14-eGFP fusion protein was targeted to the membrane and nucleus, and the GFP was ubiquitously expressed throughout the cell of *N. benthamiana* plants, suggesting that the StCDPK14 protein was membrane- and nucleus-associated (Figure 4C).

### Verification of *StCDPK14*-Interference Plants

**FIGURE 5:**
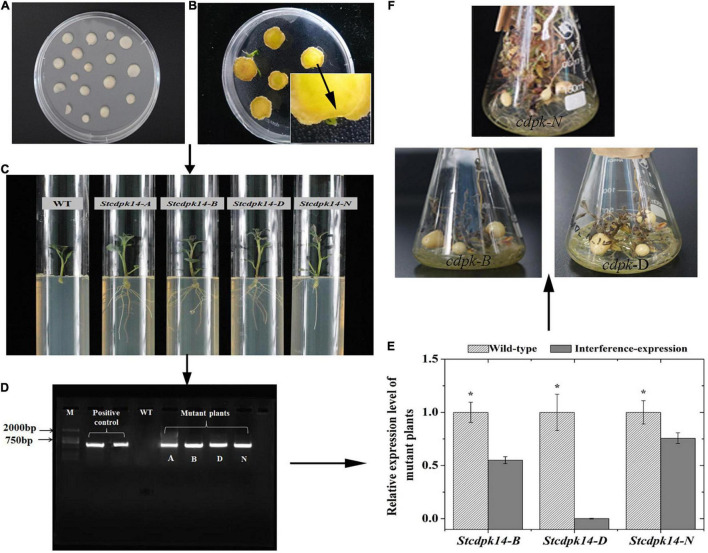
Regeneration and verification of *StCDPK14* transgenic potato tubers. (**A**: Callus formation on the center of tuber slices; **B**: Shoot formation; **C**: The roots selection of transgenic plant; **D**: PCR identification of genomic DNA from Kana-resistant potato plant (M: DL2000 marker); **E**: Quantitative RT-PCR analysis of *StCDPK14* in the transgenic tubers; **F**: Growth of transgenic tuber). Asterisk indicates a significant difference (*P* < 0.05) between wild-type and interference-expression plants.

We successfully generated transgenic plant and tubers as shown in Figure 5. The amplification of expected 600 bp DNA fragment using NTP II gene specific primers appeared in four lines (*StCDPK14-A, B, D, N*), but not in wild-type line. Further confirmation of *StCDPK14* expression in the successfully interference plants (*StCDPK14-B*, *D*, *N*) indicated that the transcript level of *StCDPK14-D* was noticeably inhibited compared to the other two lines.

### *StCDPK14* Was Successfully Repressed in Transgenic Potato Tubers

**FIGURE 6:**
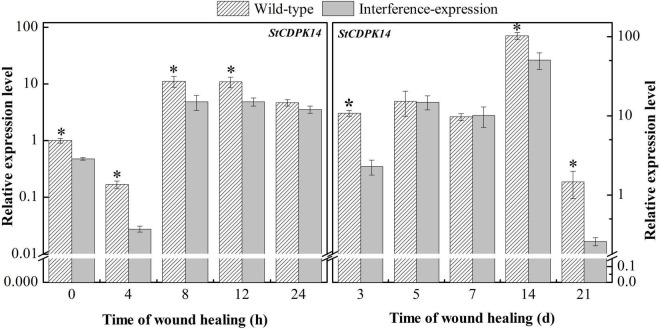
The relative expression of *StCDPK14* in transgenic tubers. The potato elongation factor 1-alpha 1 (*efla)* gene was used as an internal control to normalize the data. The mean (±SD) represents the value of three replicates. Asterisks indicate statistical significance (*P* < 0.05).

To determine whether a decrease in *StCDPK14* expression occurred throughout the healing stage in transgenic potato tubers, the expression of wild-type and interference-expression tubers was compared. *StCDPK14* displayed a similar expression tendency in wild-type and interference-expression tubers; the interference-expression tubers had a lower transcript level during the first 24 h and the late stage of healing (Figure 6). Obviously, the expression of *StCDPK14* peaked at 8 h of healing, which was 56.7% lower than that of the wild type. However, another peak of *StCDPK14* expression level was observed at 14 days of healing and was 50.6% lower in interference-expression tubers than the wild-type tubers. These data indicated that the *StCDPK14* in interference-expression potato tubers was suppressed.

### The Interference of *StCDPK14* Affected the Expression of *StRbohs*, O_2_^–^, and H_2_O_2_ Accumulation in Transgenic Tubers

**FIGURE 7:**
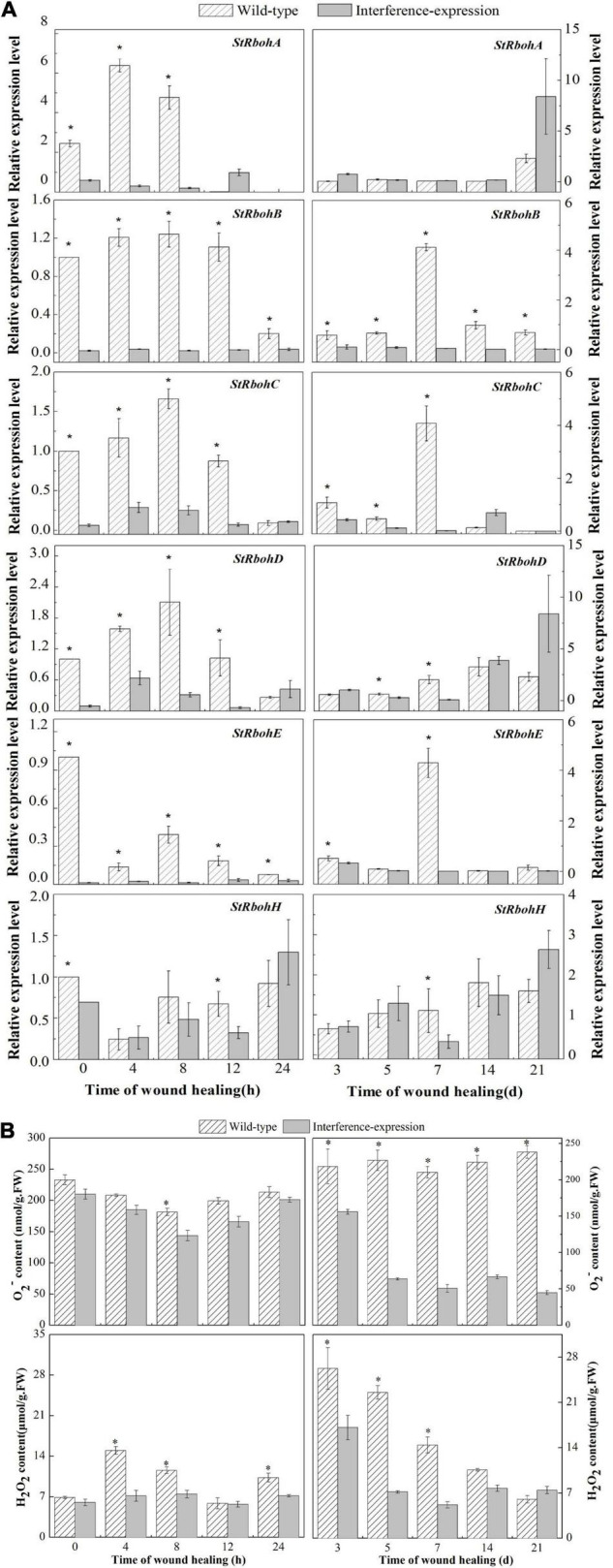
Relative expression of *StRbohs* genes and O_2_^–^ and H_2_O_2_ content during healing in potato tubers. **(A)** The relative expression of *StRbohs* genes in transgenic tubers. **(B)** The O_2_^–^ and H_2_O_2_ content during healing in transgenic tubers. The potato elongation factor 1-alpha 1 (*efla)* gene was used as an internal control to normalize the data. The mean (±SD) represents the value of three replicates. Asterisks indicate statistical significance (*P* < 0.05).

To illustrate whether the interference-expression of *StCDPK14* impacted the Rbohs genes, the expression levels of *StRbohs (A–H)* in transgenic tubers were also examined (Figure 7A). The interference-expression of *StCDPK14* resulted in a marked decrease in the transcript levels of *StRbohs* during wound healing, including the early 24 h of healing. The expression of *StRbohA-H* in the wild type reached maximum levels ranging from 0.5 to 5.6 during wound healing, whereas the expression in the *StCDPK14* transgenic tubers was lower than that. Interestingly, the inhibition effect on *StRbohB* is the most obvious throughout the whole period of healing, especially within the first 24 h of healing, which was remarkably inhibited by 13.1, 9.7, and 7.4-fold at 4, 8, and 12 h of healing under the interruption of *StCDPK14*. However, the expression of *StRbohA/C/D*/E/H (except for *StRbohB*) was not significantly inhibited in the later stage of healing. This result indicated that the interference-expression of *StCDPK14* affected the *StRbohs* expression in the early healing stage of potato tubers and the effect on *StRbohB* was the most significant.

In the *StCDPK14* transgenic line, a gradually reduced O_2_^–^ content along with wound healing was observed, and the control showed a gradually increased tendency, instead (Figure 7B). The maximum difference in O_2_^–^ content between interference-expression and wild-type tubers was displayed at 21 days of healing, which was 81.2% lower than the wild type. However, the H_2_O_2_ content in the two groups peaked at 4 h and 3 days of healing. After *StCDPK14* was interrupted, H_2_O_2_ levels showed a notable decrease compared to that of the wild type. A significant decrement of 52.8 and 35% lower than the wild type was revealed at 4 h and 3 days of healing, respectively. These results indicated that the interference-expression of *StCDPK14* suppressed O_2_^–^ and H_2_O_2_ production during healing in tubers.

### StCDPK14 Affected Suberin Deposition in Potato Tubers

**FIGURE 8:**
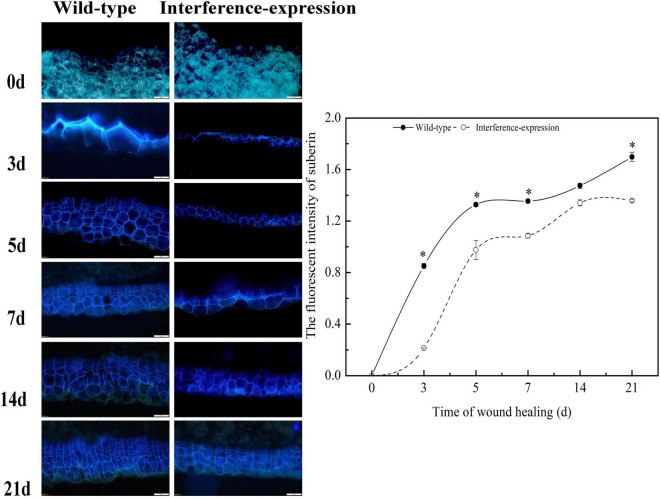
Effect of *StCDPK14* on suberin deposition at wound sites of tubers. Blue fluorescence indicated the suberin accumulation in wounded potato tubers. Bar = 200 μm. The right chart is the fluorescent intensity of suberin of wild-type and interference-expression tubers (Data are presented as mean ± SD, *n* = 3, *P < 0.05).

To evaluate the suberin deposition on the wounded surface of tubers, observation of tuber sections was performed by fluorescent microscopy. The results revealed that the interruption of *StCDPK14* had a distinct effect on suberin deposition (Figure 8). The captured fluorescent signal meant a deposition of suberin in the wild-type control and interference-expressed tubers during healing. Obviously, the suberin deposition in the *StCDPK14*-interference tubers was less than that of the wild-type tubers at each time point of tuber healing, and the maximum difference was observed at 14 days. Thus, the interference of *StCDPK14* caused a reduction of suberin deposition in wounded tubers.

### StCDPK14 Interacted With StRbohB

**FIGURE 9:**
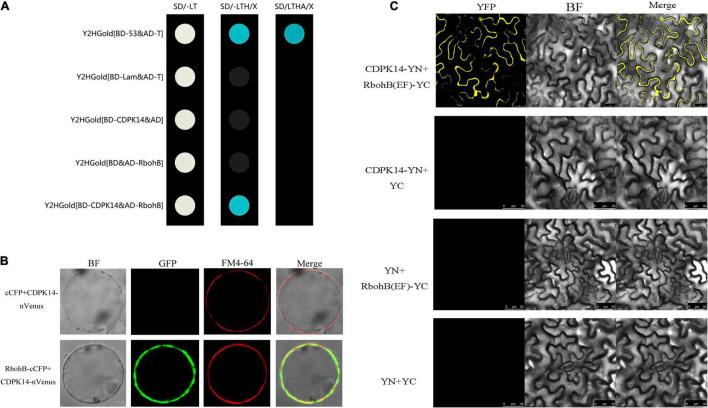
Yeast-two-hybrid assay and a bimolecular fluorescent complimentary (BiFC) assay of interactions between StCDPK14 and StRbohB. **(A)** Yeast-two-hybrid assay of interactions between StCDPK14 and StRbohB. The pGBKT7-53 + pGADT7-T was set as a positive control and pGBKT7-Lam + pGADT7-T, pGBKT7-StCDPK14 + pGADT7, and pGADT7-StRbohB + pGBKT7 were set as negative control, respectively. **(B)** A BiFC assay of interactions between StCDPK14 and StRbohB. Full-length StCDPK14 protein was fused to N-terminal Venus (nVenus-CDPK14), and full-length StRbohB was fused to C-terminal CFP (StRbohB-cCFP). The expression of cCFP/nVenus-CDPK14 was used as control. Bar = 10 μm. **(C)** A BiFC assay shows interactions between StCDPK14 and the EF-hand motifs of StRbohB in tobacco leaf epidermal cells. StCDPK14 and the EF-hand motifs of StRbohB were fused with the N and C termini of YPF. StCDPK14 with C-terminal alone, StRbohB (EF) with N-terminal alone and only C, N-terminal was used as the negative control. Bar = 20 μm.

The protein interaction prediction showed that StCDPK14 could interact with StRbohA, StRbohB, or StRbohC, namely, these proteins might be the substrates of StCDPK14 (Figure 4B). Our previous transcriptomic analysis indicated *StCDPK14* and *StRbohB* were induced the most after BTH treatment (Supplementary Table 1). And above results showed that the transcript level of *StRbohB* was also dramatically reduced the most when *StCDPK14* was interrupted. Therefore, a yeast-two-hybrid screen between StCDPK14 and StRbohB (PGSC0003DMG400024754) was performed to identify the interaction. The full length of StCDPK14 was fused to the GAL4 DNA binding domain of the bait vector to create the construct. For the verification of the interaction with StRbohB, the coding regions of each protein were introduced into the GAL4 activation domain of the prey vector. After the co-transformation into the Y2HGold yeast strain, the protein-protein interaction between them was reconstructed. The yeast-two-hybrid result showed that the fusion protein of StCDPK14 with StRbohB was expressed in medium lacking Leu-Trp-His and blue colonies are observed on medium with addition of X-a-gal (Figure 9A), suggesting that StCDPK14 interacted with StRbohB.

Moreover, a BiFC assay was selected to further verify the interaction of StCDPK14 with StRbohB. StCDPK14 specifically interacted with StRbohB and localized to the plasma membrane (Figure 9B). Rboh was reported to be phosphorylated at N-terminal extension with EF-hand motifs by CDPK. To further confirm the interaction between StCDPK14 and EF-hand motifs of StRbohB, another BiFC assay was performed to identify the interaction. As expected, StCDPK14 specifically interacted with the EF-hand motifs of StRbohB (Figure 9C), which indicated that the potential interaction sites exist at N-terminal of StRbohB.

## Discussion

Ca^2+^, as a unique second messenger in plants, plays a particularly important role in signal transduction and is involved in various biological processes (Liu Y. et al., 2017), and also required for defense response against mechanical wounding (Kawano and Muto, 2000; Toyota et al., 2018). Ca^2+^ signal originates through appropriate environmental stresses, which is transferred into the nucleus where the related genes could involve in transcription activity (Swarbreck et al., 2013). In the process of Ca^2+^ signal generating, Ca^2+^ channel proteins that are located in plasma membrane or intracellular membrane of certain organelles such as vacuole, mitochondria, chloroplast, and endoplasmic reticulum are activated and results in Ca^2+^ influx, leading to an increase of Ca^2+^ concentration (Chinnusamy et al., 2004). In the current study, the concentration of cellular Ca^2+^ that mainly located in subcellular structure of cells at wounded sites (Figure 1) is induced after BTH treatment (Figure 2), which is similar to the report that cytosolic Ca^2+^ concentration and ROS generation in tobacco suspension culture are induced by SA, an analog of BTH (Kawano and Muto, 2000). SA was also reported to induce Ca^2+^ movement and leads to a higher cytosolic Ca^2+^ level and antioxidant activities in grape plant (Wang and Li, 2006). Hence, we speculate that BTH could activate Ca^2+^ channels and induce the Ca^2+^ influx in healing tissues, then the increasing intracellular Ca^2+^ concentration provokes Ca^2+^ binding to CDPK motif and regulates CDPK activity.

In plants, the stimulus-associated [Ca^2+^]cyt fluxes are perceived and transduced by Ca^2+^-binding proteins that could relay into the downstream response processes leading to changes of genes in transcriptional activity and phosphorylation cascades (Perochon et al., 2011). These proteins including CDPKs contain a CaM-like domain of Ca^2+^- binding in their C-terminal (Harmon et al., 2001). Once the Ca^2+^ is bound to CaM-like domain, the CDPK activity could be activated (Parvathy, 2018). CDPKs belong to a multigene family in many plants, and 23 typical CDPKs have been isolated in potato. Our data showed that most of the members, including *StCDPK14*, are significantly elevated in BTH-treated tissues (Figure 3), which is in agreement with the upregulated transcript levels of *MdCDPK1*/*4*/*5*/*7*/*21* noticed by acibenzolar-S-methyl (ASM) (Hou et al., 2021) and *SlCDPK1-29*, except for *SlCDPK7* and *SlCDPK14*, in response to exogenous SA in tomatoes (Hu et al., 2016). In banana plants, eight different CDPK proteins in BTH-sprayed plants are similarly induced to accumulate to a higher level (Cheng et al., 2018). The expression levels of CDPKs in grape and strawberry fruits treated with BTH or SA were all elevated (Landi et al., 2014; Zhang et al., 2015). In addition, in the response of *LeCDPK2* to SA, the transcript of *LeCDPK2* was also enhanced (Chang et al., 2009). Thus, the increased *StCDPKs* transcript levels may be reflected by the Ca^2+^ signal caused by BTH-inducible Ca^2+^ concentration in the cytosol, which trigger the related gene expression in the nucleus and allow them to function as Ca^2+^ sensors (Kolukisaoglu et al., 2004). In the present study, the BTH-induced *StCDPK14* showed the most significant transcriptional level. Herein, we propose that *StCDPK14* might participate in regulation of the BTH-induced healing process of potato tubers.

*Solanum tuberosum* calcium-dependent protein kinase 14 has been characterized and predicted in group II a (Fantino et al., 2017), and shows a high similarity to the species of *AtCDPK29*, *NtCDPK19*, and *SlCDPK29* species (Figure 4A), among which *AtCDPK29* has been found to be involved in disease resistance to *Pseudomonas syringae* pv. *tomato* (*Pst*) DC3000 (Wang et al., 2015). Meanwhile, the *StCDPK14* is predicted to harbor both myristoylation and palmitoylation motifs at the N-terminus, which has been reported to play a critical role in facilitating protein-protein interaction (Xu et al., 2015). The results in this work found that the GFP-tagged *StCDPK14* protein was predominantly localized to the plasma membrane and nucleus by analysis of transient expression in the *N. benthamiana* leaves (Figure 4C). This specific subcellular localization may confer loose membrane association to target proteins and provides unique roles in regulating different cellular functions (Simeunovic et al., 2016).

The Ca^2+^ signals are essentially a kind of chemical code and the decoding process requires Ca^2+^ sensors, such as CDPK. Then, the information encoded in the Ca^2+^ signature is translated into a phosphorylation event of the target protein (Hashimoto and Kudla, 2011). It is reported that Rbohs are *in vitro* substrates of CDPK that can decode Ca^2+^ signatures into phosphorylation of Rboh proteins (Giammaria et al., 2011; Hashimoto and Kudla, 2011; Wang et al., 2015). Based upon this, for a further insight into demonstrating the hypothesis that *StCDPK14* was involved in the healing event by regulating the activity of RBOH proteins, we successfully obtained the interference-expressing of *StCDPK14* plants and tubers (Figure 5). The assay of gene expression revealed that interference-expression of *StCDPK14* resulted in a decline levels of its own transcript and *StRbohA*-*H* throughout the wound healing period in tubers (Figures 6, 7A), which might account for the involvement of *StCDPK14* in the activation process of RBOH during wound healing in potato tubers. Previous studies also documented the relationship between CDPKs and Rboh proteins, that is, the phosphorylation of StRboh by StCDPK (Gromadka et al., 2018) and the oxidative burst resulting from increased CDPK expression in potato (Polkowska-Kowalczyk et al., 2004). Therefore, an assumption was that StCDPK14 activated the Rboh activity *via* a specific event to regulate ROS generation during wound healing of potato tubers.

In the current study, putative interaction proteins were verified by using yeast-two-hybrid and a BiFC assay, which was allowed to detect the protein-protein interactions and furthermore can be used to observe the subcellular localization of the interacting proteins (Walter et al., 2004). The results showed that StCDPK14 interacted with StRbohB at the membrane (Figures 9A,B), which is corresponded with the *in silico* protein interaction network prediction (Figure 4B), indicating that StRbohB proteins were the targets and action substrates of StCDPK14. Interestingly, StCDPK14 was found to interact with EF-hand motifs of StRbohB at N-terminal (Figure 9C). Kobayashi et al. (2007) reported that StRbohB N-terminus region exits potential phosphorylation sites for CDPK5, and the Rboh contains N-terminal EF-hand that used to bind Ca^2+^ for full activation (Oda et al., 2010). It has also been reported that AtRbohD was activated by ionomycin-induced cytosolic Ca^2+^ influx through dual mechanisms synergistically: by changing conformation in EF-hand region, and by modification event at N-terminal through CDPKs (Kaur et al., 2014). Therefore, we speculated that there are probably potential phosphorylation sites at N-terminal of StRbohB for StCDPK14 and a indirectly phosphorylation reaction in a Ca^2+^-dependent manner between StRbohB and StCDPK14 occurs during wound healing of tubers, or the Ca^2+^-binding to EF-hand of StRbohB N-terminus directly leads to the activation of StRbohB. However, the oxidative burst downstream by Rbohs is a common immune response to disease resistance, which is intimately tied to CDPK (Polkowska-Kowalczyk et al., 2004). A series of CDPKs including *StCDPK5* (Kobayashi et al., 2007; Gao et al., 2013), *AtCDPK5* (Dubiella et al., 2013), *BnaCDPK2* (Wang et al., 2018), and several *BrrCDPKs* associate with Rbohs, further indicating the activation of Rbohs mediated by CDPK regulates the ROS production and leads to an oxidative burst when plants suffer from biotic stress (Bhattacharjee, 2005).

The generation of ROS, especially H_2_O_2_ derived from O_2_^–^ that mainly generated by an NADPH oxidase system, was thought to be required in the polymerization of phenolic domain of suberin (Razem and Bernards, 2003; Lulai et al., 2016). The homolog StRbohA in potato has been demonstrated to involve the wound healing of tubers (Kumar et al., 2007; Jiang et al., 2020). The suberin deposition during wound healing in potato tubers is a specific polymerization process that requires the involvement of H_2_O_2_ (Kumar et al., 2007). A decrease of O_2_^•–^ and H_2_O_2_ content was determined in the *StCDPK14* interference-expression transgenic tubers in the current results (Figure 7B). Moreover, less suberin deposition in interference-expression tubers was also observed. These findings indicated that interference-expression of *StCDPK14* might affect the Rbohs activity by altering the expression pattern and reduce O_2_^•–^ and H_2_O_2_ production, leading to a decrement in suberin deposition (Figure 8). Hence, we infer that *StCDPK14* might play a positive role in manipulating O_2_^•–^ and H_2_O_2_ generation during suberin formation in potato tubers induced by BTH.

**FIGURE 10:**
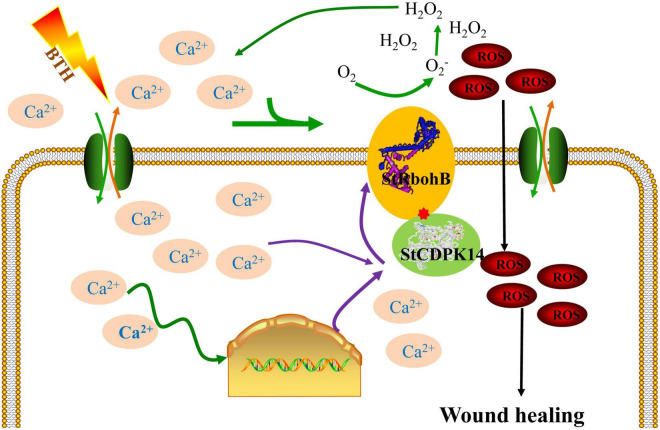
Integration of regulatory mechanisms of StCDPK14 involving in the wound healing of potato tubers treated with BTH. Proposed regulations are shown in the scheme as discussed in this article. The elicitor BTH induced the Ca^2+^ influx in the cytosol and triggers the relaying of Ca^2+^ signal in cells. Once the Ca^2+^ signal generated, CDPK (Ca^2+^ sensor) activity was activated by Ca^2+^ binding to EF-hand domain. Subsequently, the downstream RbohB activity was activated *via* interacting with CDPK and produced reactive oxygen species (ROS), which was tied to the wound healing as a signal molecule and oxidative cross-linking the precursors of healing tissues.

Taken together, *StCDPK14*, a gene encoding a CDPK from *S. tuberosum*, is provoked by BTH-induced Ca^2+^ influx. Then, the activated StCDPK14 further interacted with downstream element StRbohB, which affects O_2_^•–^ and H_2_O_2_ generation. Therefore, *StCDPK14* was considered to involve the wound healing of potato tubers by regulating Rboh-dependent ROS generation (Figure 10). The interaction between StCDPK14 and StRbohB allows further insight into the diverse roles and potential mechanism of StCDPK during wound healing. Meanwhile, the knowledge of StCDPKs signaling pathways in response to wound healing induced by elicitors was expanded. It will be essential for future work to clarify the possibility of StRbohB phosphorylated by StCDPK14 in ROS regulating the wound healing of potato tubers.

## Data Availability Statement

The datasets presented in this study can be found in online repositories. The names of the repository/repositories and accession number(s) can be found in the article/Supplementary Material.

## Author Contributions

HJ and LM performed the experimental work, data analysis, and manuscript preparation. YB and Y-CL were responsible for research outline and experimental design. Y-YR, J-WY, and H-JS guided the transgenic technology guidance. DP was responsible for experimental design and language revisions. All authors contributed to the article and approved the submitted version.

## Conflict of Interest

The authors declare that the research was conducted in the absence of any commercial or financial relationships that could be construed as a potential conflict of interest.

## Publisher’s Note

All claims expressed in this article are solely those of the authors and do not necessarily represent those of their affiliated organizations, or those of the publisher, the editors and the reviewers. Any product that may be evaluated in this article, or claim that may be made by its manufacturer, is not guaranteed or endorsed by the publisher.

## Footnotes

1
https://www.ncbi.nlm.nih.gov/

2
http://smart.embl-heidelberg.de

3
http://web.expasy.org/myristoylator/

4
http://csspalm.biocuckoo.org/

5
http://string-db.org/

## Supplementary Material

Supplementary Table 1 The FPKM value of *StCDPKs* and *StRbohs* were upregulated after BTH treatment in the transcriptomic analysis.
