# Supplementary material for: The Interaction Between StCDPK14 and StRbohB Contributes to Benzo-(1, 2, 3)-Thiadiazole-7-Carbothioic Acid S-Methyl Ester-Induced Wound Healing of Potato Tubers by Regulating Reactive Oxygen Species Generation
Source: Front Plant Sci. 2021 Nov 15;12:737524. doi: 10.3389/fpls.2021.737524 (PMC8634758; doi:10.3389/fpls.2021.737524)
Supplement: Supplementary Table 1 — The FPKM value of StCDPKs and StRbohs were upregulated after BTH treatment in the transcriptomic analysis. [file Table_1.docx]

| PGSC accession number  (CDPK & RBOH) | Control-5d | | | BTH-5d | | | NCBI accession number |
| --- | --- | --- | --- | --- | --- | --- | --- |
|  | T1 | T2 | T3 | T7 | T8 | T9 |  |
| PGSC0003DMG400003213 | 23.18957 | 22.92016 | 23.40741 | 45.27569 | 41.67213 | 42.24487 | LOC102581410 |
| PGSC0003DMG400003564 | 8.354246 | 8.972903 | 9.16802 | 25.79043 | 24.7315 | 23.36125 | LOC102590362 |
| PGSC0003DMG400008149 | 70.73581 | 66.25906 | 65.65436 | 167.8375 | 161.5734 | 182.8347 | LOC102585161 |
| PGSC0003DMG400009883 | 9.351418 | 9.648767 | 9.577284 | 40.42025 | 41.87118 | 36.66486 | LOC102580468 |
| PGSC0003DMG400010059 | 0.752461 | 0.676309 | 0.608342 | 1.996657 | 2.395213 | 2.244767 | LOC102588824 |
| PGSC0003DMG400013183 | 126.55 | 133.7389 | 137.1163 | 235.4036 | 222.4719 | 244.1997 | LOC102591452 |
| PGSC0003DMG400014797 | 8.30724 | 8.48364 | 8.448672 | 14.74233 | 18.42776 | 15.0675 | LOC102582093 |
| PGSC0003DMG400022318 | 21.89094 | 20.41069 | 17.12341 | 49.03144 | 46.09973 | 47.26363 | [XM_006346152.2](https://www.ncbi.nlm.nih.gov/nucleotide/XM_006346152.2?report=genbank&log$=nucltop&blast_rank=1&RID=E59XR882013) |
|  | | | | | | | |
| PGSC0003DMG400024754 | 14.65102 | 14.74415 | 13.0137 | 52.17402 | 52.94526 | 54.95255 | LOC102603596 |
| PGSC0003DMG400014168 | 186.9579 | 172.7778 | 158.4283 | 392.2085 | 360.6433 | 441.9645 | LOC102598898 |
