# Supplementary figures and images for: The Interaction Between StCDPK14 and StRbohB Contributes to Benzo-(1, 2, 3)-Thiadiazole-7-Carbothioic Acid S-Methyl Ester-Induced Wound Healing of Potato Tubers by Regulating Reactive Oxygen Species Generation
Source: Front Plant Sci. 2021 Nov 15;12:737524. doi: 10.3389/fpls.2021.737524 (PMC8634758; doi:10.3389/fpls.2021.737524)

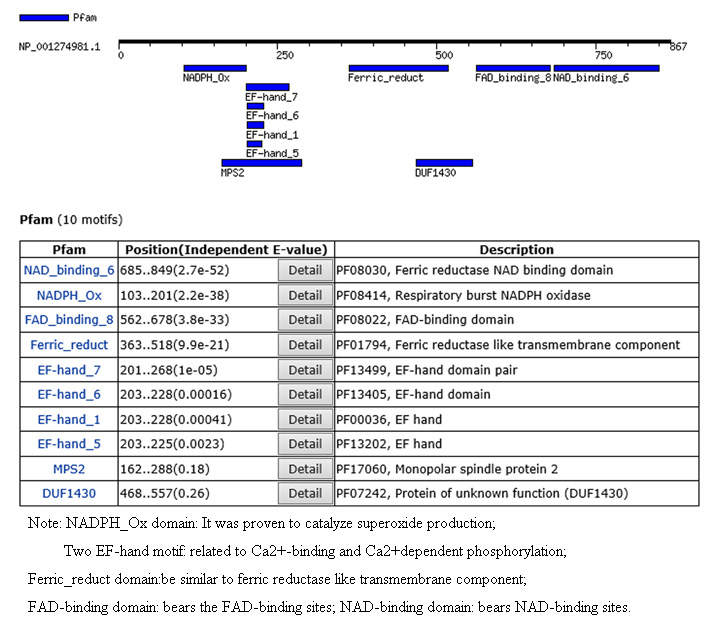

Supplement: Supplementary file 2 [file Image_1.JPEG]
